# My Virtual Parent, Adult, and Child: Simulating Ego-States in Transactional Analysis with LLM

**DOI:** 10.1192/j.eurpsy.2025.1421

**Published:** 2025-08-26

**Authors:** A. M. Bukinich, A. M. Gerashenko, G. D. Vzorin, A. M. Konovalova

**Affiliations:** 1 Faculty of Psychology, Lomonosov Moscow State University; 2 Department of Pedagogy and Medical Psychology, Sechenov University, Moscow, Russian Federation

## Abstract

**Introduction:**

Recent advancements in large language models (LLMs) demonstrate their ability to perform verbal generalization and simulate agency, opening new possibilities for innovative applications in psychotherapy. One promising avenue is the integration of LLMs into Eric Berne’s Transactional Analysis, a psychotherapeutic approach that conceptualizes human personality through three distinct ego-states: Parent, Adult, and Child.

**Objectives:**

To investigate the potential of LLMs in Transactional Analysis.

**Methods:**

LLM capabilities described in the literature were compared with demands derived from the theory.

**Results:**

By examining the interactions among ego-states in Transactional Analysis, individuals can become aware of their behavior patterns and modify ineffective ones, fostering healthier relationships and improving social adaptation. Incorporating LLMs into Transactional Analysis offers a novel tool for psychotherapeutic interventions. Therapists could utilize AI to help clients recognize emotional experiences and identify maladaptive interaction patterns. By simulating the Parent, Adult, and Child ego-states, an LLM could engage in dialogues that reflect these internal states, allowing the client to explore and better understand their emotional reactions and needs. This interaction would enable clients to actively engage with different parts of their personality, promoting self-awareness and providing valuable insights into the causes of relational difficulties. While the potential benefits are clear, further research is needed to assess the practical effectiveness of integrating LLMs into Transactional Analysis. Empirical studies should investigate the degree to which LLMs can accurately simulate ego-states and contribute to positive therapeutic outcomes. Additionally, ethical considerations must be addressed to ensure that the application of AI in psychotherapy is both responsible and beneficial for clients.

**Conclusions:**

The proposed approach fosters a deeper understanding of internal conflicts and provides a structured, controlled environment where clients can work through problematic situations.

**Disclosure of Interest:**

None Declared

